# Possible transfusion-related acute lung injury (TRALI) in cardiac surgery patients

**DOI:** 10.3325/cmj.2014.55.138

**Published:** 2014-04

**Authors:** Tajana Zah-Bogović, Jasna Mesarić, Pero Hrabač, Višnja Majerić-Kogler

**Affiliations:** 1 Department of Anesthesiology, Resuscitation and Intensive Care, University of Zagreb, School of Medicine, Zagreb University Hospital Center, Zagreb, Croatia; 2 Agency for Quality and Accreditation in Health Care and Social Welfare, University of Zagreb, School of Medicine, Zagreb, Croatia; 3 Croatian Institute for Brain Research, University of Zagreb, School of Medicine, Zagreb, Croatia; 4 University of Zagreb, School of Medicine, Zagreb, Croatia

## Abstract

**Aim:**

To determine the incidence of possible transfusion-related acute lung injury (TRALI) and related risk factors in cardiac surgery patients.

**Methods:**

A single-center prospective cohort study was conducted from January 2009 to March 2010 at the Zagreb University Hospital Center, Croatia. Patient-, transfusion-, and surgery-related data were collected. The study included 262 patients who were observed for respiratory worsening including measurements of arterial oxygen saturation (SaO_2_), fraction of inspired oxygen (FiO_2_), and partial pressure of arterial oxygen (PaO_2_). Possible TRALI was defined according to the Toronto Consensus Conference definition broadened for 24-hour post-transfusion. This cohort was divided in two groups. TRALI group included 32 participants with diagnosis of TRALI and the control group included 220 patients with or without respiratory worsening, but with no signs of ALI.

**Results:**

Possible TRALI was observed in 32 (12.2%) patients. Compared with the control group, possible TRALI patients had higher American Association of Anesthesiology scores, higher rate of respiratory comorbidity (43.8% vs 15.5%), and required more red blood cells (median 4, range [2.5-6] vs 2 [1-3]), plasma (5 [0-6] vs 0 [0-2]), and platelet units (0 [0-8] vs 0 [0-0]) (*P* < 0.001 all). Risk factors for possible TRALI were total number of transfused blood units (odds ratio [OR] 1.23; 95% confidence interval [CI] 1.10-1.37) and duration of cardiopulmonary bypass (OR 1.08; 95% CI 1.05-1.11). Post-transfusion PaO_2_/FiO_2_ ratio was significantly decreased in possible TRALI patients and significantly increased in transfused controls without acute lung injury.

**Conclusion:**

We observed a higher rate of possible TRALI cases than in other studies on cardiac surgery patients. Serial monitoring of PaO_2_/FiO_2_ ratio and detection of its post-transfusion worsening aids in identification of possible TRALI cases.

Patients undergoing cardiac surgery often require supportive blood transfusion therapy. The rates of allogeneic blood transfusions are high, varying from 40% to 90% (1-3). This may be attributed to the fact that anemia is an independent risk factor for higher morbidity and mortality and that cardiac surgery patients may have a higher transfusion threshold (4,5). On the other hand, recent studies have shown a worse outcome in patients who received transfusion (2,6). Significant outcome improvement has been clearly demonstrated after implementation of the blood use guidelines of the American Association for Thoracic Surgery (7). In addition, due to the acquired disorder of hemostasis with the use of cardiopulmonary bypass, many patients receive plasma and platelet transfusion.

Although transfusion therapy can be lifesaving, it may also be life-threatening if patients develop transfusion-related lung injury (TRALI). TRALI is a clinical syndrome characterized by the onset of respiratory distress temporally related to transfusion of any blood component. TRALI is considered the leading cause of transfusion-related morbidity and mortality (8-10). This serious adverse event of transfusion is underreported and underestimated (11-14). One of the main reasons is reduced awareness of TRALI among clinicians. The definition seems straightforward but clinical presentation of TRALI is indistinguishable from acute respiratory distress syndrome (ARDS) due to other causes. Cases of TRALI remain unnoticed or misdiagnosed because of a diversity of its clinical symptoms, as well as the absence of specific disease markers and diagnostic tests (15).

When applying standard definitions of TRALI, a unique challenge are patients undergoing cardiac surgery (10,16-18). Cardiac surgery carries multiple risk factors for ARDS/ALI and often leads to TRALI being misdiagnosed. To identify such cases, the term possible TRALI has been coined, which allows for the presence of alternative risk factors for acute respiratory worsening (10).

There is limited clinical evidence as to why only a subset of cardiothoracic surgical patients experience worsening of respiratory function after exposure to blood transfusions. There is also a knowledge gap on respiratory complications occurring post-transfusion (15,19). In the past, TRALI has been only sporadically reported in Croatia and so far there are no published clinical studies. Therefore, we aimed to evaluate whether transfusion was associated with a worsening respiratory status in cardiothoracic surgical patients in Croatia. Previous studies provided some data on the prevalence and risk factors for classic TRALI, which by definition develops within 6 hours post-transfusion (10). Since some studies have shown that critically ill patients are at an increased risk of ALI up to 72 hours post-transfusion, we investigated whether the same was true for TRALI (19). This is also the first study that investigated the incidence of TRALI for a period up to 24 hours post-transfusion to identify not only the classic but also the delayed possible TRALI cases. Respiratory worsening is routinely recorded in intensive care unit (ICU) and passive reports of TRALI are generally imprecise. We used a prospective study design so we could connect all measured respiratory deteriorations with previous transfusions and exclude other differential diagnoses to identify possible TRALI. In order to better identify connections of respiratory worsening with previous transfusions, consecutive measurements of respiratory status (including measurements of arterial oxygen saturation [SaO_2_]), fraction of inspired oxygen [FiO_2_], partial pressure of oxygen in arterial blood [PaO_2_]) were performed in a controlled setting, thus facilitating identification of possible TRALI cases.

## Patients and methods

**Figure 1:**
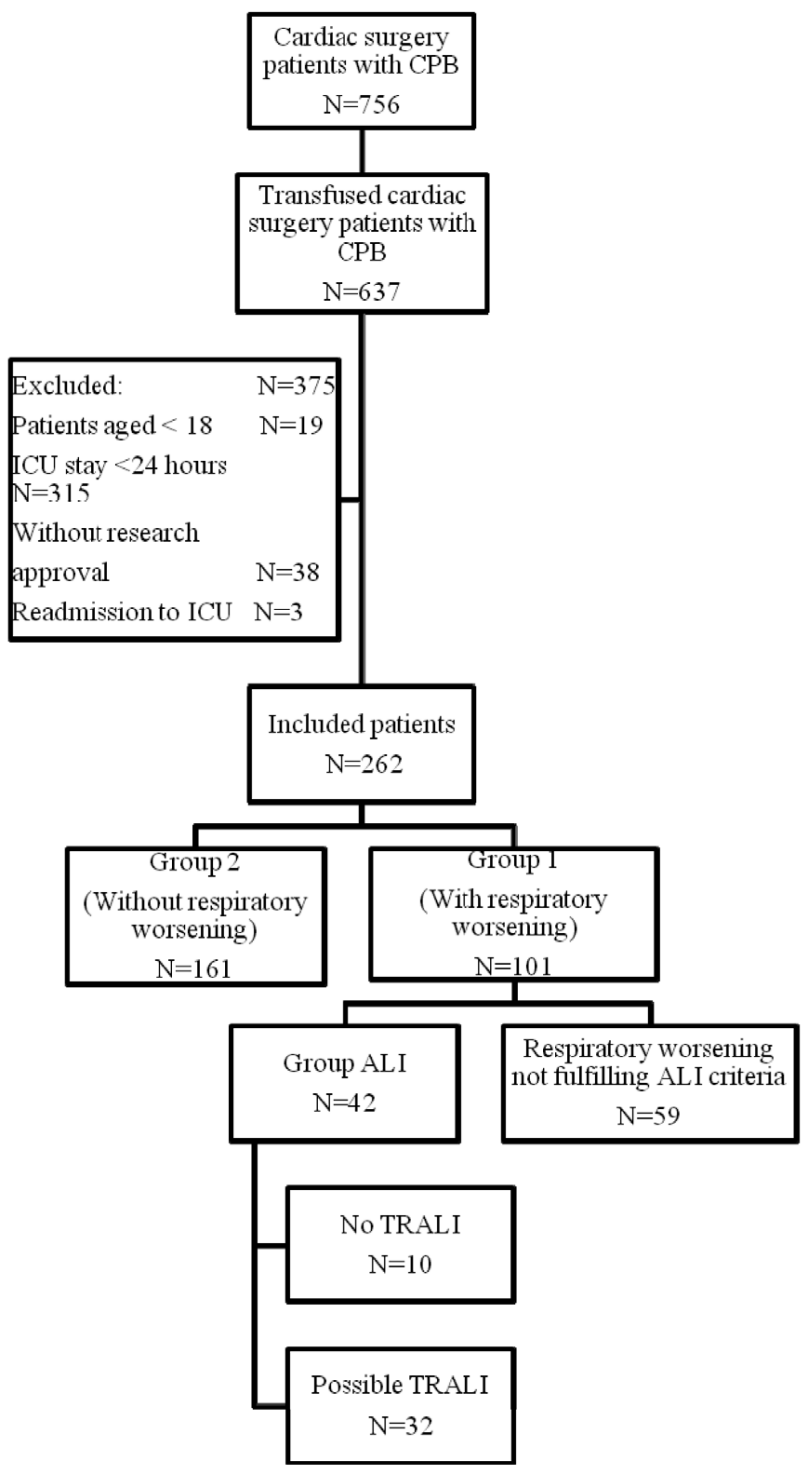
Flow diagram of patient groups.

A total of 756 consecutive patients who had undergone cardiothoracic surgery with cardiopulmonary bypass at the Zagreb University Hospital Center were screened for inclusion criteria between January 2009 and March 2010. Exclusion criteria were no transfusion, age <18, ICU stay <24 hours, no approval for the study, and ICU readmission (Figure 1). The study protocol was approved by the hospital Ethics Committee and a written informed consent was obtained from each patient before the surgery.

Our cohort included 262 transfused patients (171 male and 91 female) who signed informed consent, had undergone cardiothoracic surgery, and had received at least one of the blood components including red blood cells (RBC), plasma, or platelets. This cohort was divided in two groups. TRALI group included 32 participants with diagnosis of TRALI and the control group included 220 patients with or without respiratory worsening, but with no signs of ALI (Figure 1). Ten patients were excluded from this group because of a possible overlap with the TRALI group. The enrolled patients were closely observed for respiratory worsening and measurements of arterial oxygen saturation (SaO_2_), fraction of inspired oxygen (FiO_2_), and partial pressure of oxygen in arterial blood (PaO_2_) were obtained during as well as immediately after the surgery, and then every 6 hours for the next 24 hours. PaO_2_ was measured from an arterial catheter blood sample. Frontal chest radiograph, apart from the routine radiographic procedures, was done in all patients in whom respiratory worsening was observed. A pulmonary artery catheter was inserted as part of the standard perioperative monitoring.

TRALI was determined according to the Toronto Consensus Conference definition (10). To be classified as TRALI, patients had to conform to two sets of criteria. First, they had to have a diagnosis of acute lung injury (ALI) (nowadays classified according to Berlin definition as mild ARDS) (20), characterized by acute onset, lack of clinical evidence of left atrial hypertension, bilateral infiltrates on frontal chest radiograph, and PaO_2_/FiO_2_ ratio ≤300 mm Hg regardless of positive end-expiratory pressure level or oxygen saturation of <90% when the patient is breathing room air. Second, exclusion of differential diagnoses was required (such as acute coronary syndrome, transfusion-associated cardiac overload, newly emerging sepsis, and pneumonia). Since an alternative ARDS risk factor (such as aspiration, pneumonia, lung contusion, drowning, sepsis, multiple-trauma, burns, acute pancreatitis, cardiopulmonary bypass [CPB], and drug overdose) is present in critically ill patients undergoing cardiac surgery with CPB, the consensus definition name for such cases is possible TRALI (10).

Conventional definition broadened with the criteria by Marik and Corwin (21) was used, including patients with preexisting respiratory problems that worsened after transfusion and expanded monitoring time to up to 24 hours after each transfusion to discover the cases of “delayed TRALI syndrome.”

The following data were collected by an electronic data tool developed for this study as a web-based application: demographic characteristics, laboratory blood values (preoperative and perioperative), and underlying patient characteristics including admission diagnosis, body mass index (BMI), American Association of Anesthesiology (ASA) score, comorbidities, data on fluid balance, ventilation time, and ICU stay.

We also collected the following transfusion-related data: type of blood components and number of transfused RBC, plasma, and platelet units. Since in Croatia female donors are excluded, all plasma units were prepared from male donors and all platelet concentrates were prepared from four pooled buffy coats and resuspended in plasma from male donors. All RBC components were leukoreduced by post-storage filtration (<1 × 10^6^ white blood cells per unit).

The following surgery-related data were collected: duration of surgery and duration of CPB.

We performed sample size calculation (22,23) and aimed to include 100 patients with respiratory worsening complying to the inclusion and exclusion criteria. All included patients were followed up until their final discharge from the ICU.

### Statistical analysis

Normality of distribution was tested by Kolmogorov-Smirnov test, and the appropriate parametric or nonparametric tests were used to analyze between-group differences. Binary logistic regression was performed to assess the impact of a number of factors on the likelihood that patients had possible TRALI. *p* values below 0.05 were considered significant. Data analysis was performed using STATISTICA version 10 (StatSoft Inc., Tulsa, OK, 2011).

## Results

Respiratory worsening was observed in 101 patients and ALI in 42 patients. Possible TRALI, monitored for up to 24 hours after transfusion, was observed in 32/262 (12.2%) patients. ALI, but with no possibility for TRALI was observed in 10 patients because of other differential diagnoses. Classic TRALI developed within 6 hours of transfusion in 22/32 possible TRALI patients and delayed TRALI (between 6 and 24 hours) in 10/32 possible TRALI patients.

### Patient-related risk factors

On admission, when compared to the control group patients who developed TRALI had a higher rate of high ASA scores, respiratory comorbidity (43.8% vs 15.5%, lower admission hemoglobin (105.5 ± 29.2 vs 125.6 ± 21.8), lower admission platelet count (169.4 ± 145.3 vs 217.5 ± 92.4), and lower admission prothrombin time (0.7 ± 0.2 vs 0.9 ± 0.2) (*P* < 0.001 all) (Table 1).

**Table 1:** Patient characteristics, clinical data, and transfusion-related acute lung injury (TRALI) risk factors in patients with TRALI and transfused controls without ALI on admission

Parameter	Transfused controls (without ALI)	Patients with possible TRALI	*P*
Age (years; mean ± SD)	66.3 ± 10.28	62.6 ± 10.85	0.064
Male sex, n (%)	138 (62.7)	24 (75.0)	0.176
Body mass index (kg/m^2^; mean ± SD)	27.9 ± 4.51	28.4 ± 4.05	0.542
ASA score, n (%):			
II-III	183 (84.7)	19 (59.4)	<0.001
IV-V	33 (15.3)	13 (40.6)	
History of comorbidity, n (%):			
cardiologic	213 (96.8)	30 (93.8)	0.382
respiratory	34 (15.5)	14 (43.8)	<0.001
urologic	22 (10.0)	6 (18.8)	0.141
hepatic	29 (13.2)	3 (9.4)	0.546
metabolic	101 (45.9)	13 (40.6)	0.575
Hemoglobin (g/L; mean ± SD)	125.6 ± 21.8	105.5 ± 29.2	<0.001
Platelet count (n ×10^9^/L; mean ± SD)	217.5 ± 92.4	169.4 ± 145.3	<0.001
Prothrombin time (mean ± SD)	0.9 ± 0.2	0.7 ± 0.2	<0.001
Duration of surgery (min; mean ± SD)	296.3 ± 57.3	444.4 ± 103.1	<0.001
Duration of CPB (min; mean ± SD)	131.3 ± 31.1	250.6 ± 51	<0.001

*Abbreviations: SD – standard deviation; CPB – cardiopulmonary bypass; ASA – American Society of Anesthesia classification.

### Surgery-related risk factors

Patients who developed possible TRALI had longer duration of surgery (444.4 ± 103.1 vs 296.3 ± 57.3) and longer duration of CPB (250.6 ± 51 vs 131.3 ± 31.13) (*P* < 0.001 both) (Table 1). Predictive value of CPB duration for the occurrence of TRALI was assessed using receiver operating characteristic (ROC) curve. ROC area under the curve (AUC) was 0.94, indicating that CPB time could be a powerful predictor of TRALI occurrence. Based on the model, the CPB cut-off value of 178 minutes had 90% sensitivity and 90% specificity in prediction of possible TRALI.

### Transfusion-related risk factors

Compared with transfused controls without ALI, patients with possible TRALI received a higher total number of blood transfused units (median 14, range [3.5-18] vs 2.5 [1.5-5]), RBC units (4 [2.5-6] vs 2 [1-3]), plasma units (5 [0-6] vs 0 [0-2]), and platelet units (0 [0-8] vs 0 [0-0]) (*P* < 0.001 all). RBC storage time did not differ between the groups (Table 2). Out of 1349 total transfused blood units, 732 (54.3%) were leukocyte-depleted RBC, 361 (26.8%) plasma, and 256 (19.0%) platelets.

**Table 2:** Transfusion data on patients with transfusion-related acute lung injury (TRALI) and transfused controls without ALI

Parameter (median, range)	Transfused controls (without ALI)	Patients with possible TRALI	*P*
Total number of transfused blood units	2.5 (1.5-5)	14 (3.5-18)	<0.001
Number of RBC transfusions (units)	2 (1-3)	4 (2.5-6)	<0.001
Mean RBC age in days (mean ± standard deviation)	7.8 ± 3.19	7.3 ± 2.37	0.394
Number of plasma transfusions (units)	0 (0-2)	5 (0-6)	<0.001
Number of platelet transfusions (units)	0 (0-0)	0 (0-8)	<0.001

*Abbreviations: RBC – red blood cell concentrates.

### Binary logistic regression

Binary logistic regression was performed to assess the impact of a number of factors on the likelihood that patients had possible TRALI. Out of the 8 independent variables assessed in the initial testing (age, sex, BMI, ASA score, respiratory comorbidity, total number of transfused blood units, duration of surgery, and duration of CPB), 5 variables showed significant results (*P* < 0.01): ASA score (odds ratio [OR] 2.79; 95% confidence interval [CI] 1.38-5.65), respiratory comorbidity (OR 4.25; 95% CI 1.93-9.36), total number of transfused blood units (OR 1.18; 95% CI 1.11-1.24), duration of surgery (OR 1.02; 95% CI 1.02-1.03), and duration of CPB (OR 1.05; 95% CI 1.04-1.07). These variables were included in the multiple regression model. Using forward and backward selection approaches, some variables were dropped from the final model, which in the end included only 2 variables (total number of blood transfused units and duration of CPB). The model containing these 2 predictors was significant (χ^2^ = 128.6, *P* < 0.001), indicating that it was able to distinguish between the patients with and without TRALI. The model as a whole explained 77.8% (Nagelkerke R^2^, null adjusted) of the variance in symptomatology, correctly classified 95.0% of cases, and demonstrated excellent goodness of fit of 0.839 (Hosmer and Lemeshow). Two variables included in the model made a unique, significant contribution to the model: total number of transfused blood units (OR 1.23; 95% CI 1.10-1.37; *P* < 0.001) and duration of CPB (OR 1.08; 95% CI 1.05-1.11; *P* < 0.001).

**Figure 2:**
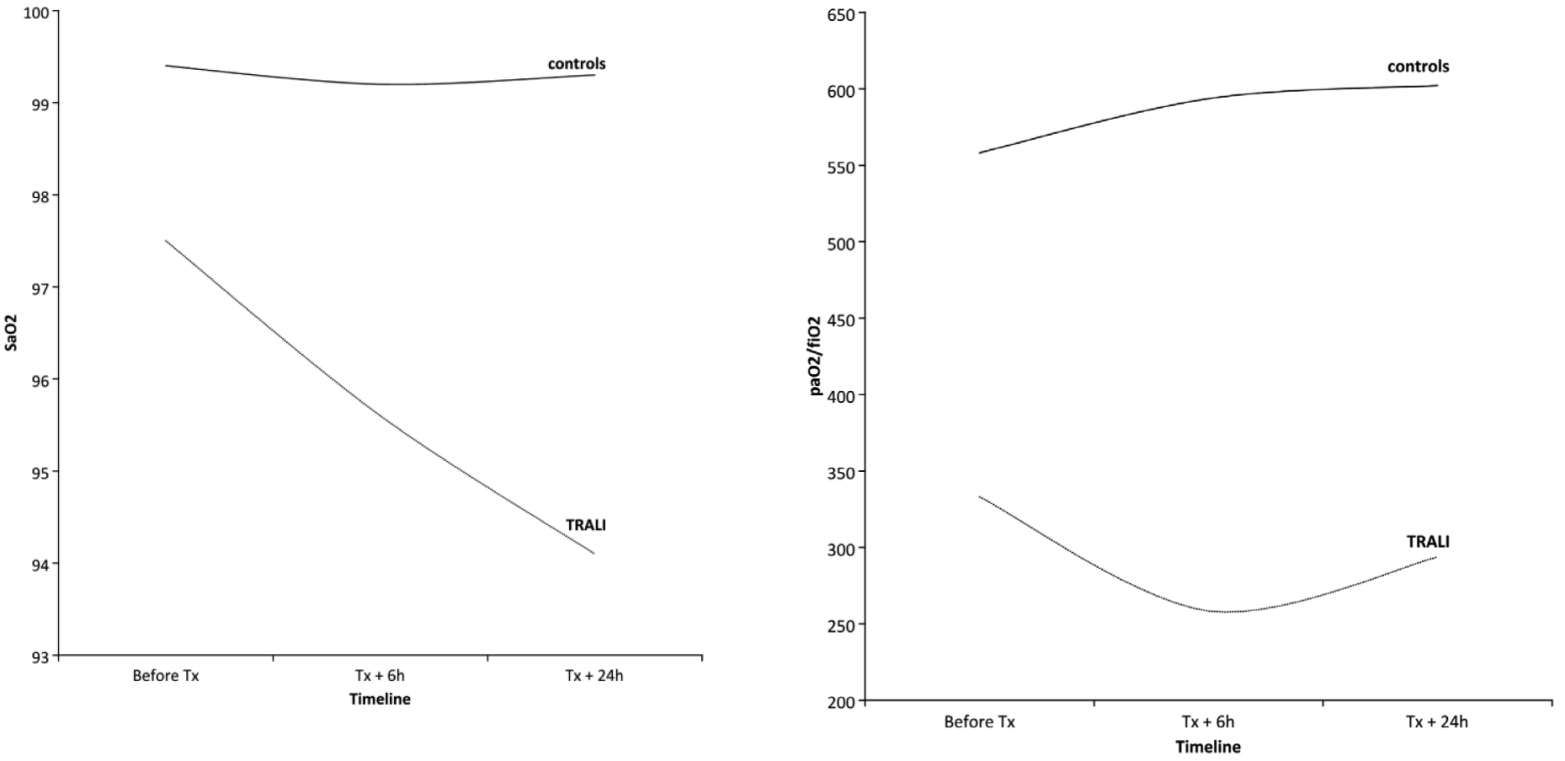
Arterial oxygen saturation (SaO_2_) (**A**) and partial pressure of arterial oxygen/fraction of inspired oxygen (PaO_2_/FiO_2_) ratio (**B**) before and after blood transfusion (Tx).

The measurement of SaO_2_ with pulse oximetry indicated that respiratory function deterioration did not prove sufficient for the ALI diagnosis as a prerequisite for clinical suspicion of possible TRALI (the value of SaO_2_ was not <90% at any point of measurement) (Figure 2). A significant decrease in the post-transfusion PaO_2_/FiO_2_ ratio was observed in possible TRALI patients, along with a significant post-transfusion increase in transfused controls without ALI (Figure 2). Possible TRALI patients had a significantly longer ICU length of stay (hours) (median 96.5, range [31.5-152.4] vs 29.1 [28.0-66.1]; *P* < 0.001).

## Discussion

The main findings of our study were as follows: 1) the incidence of possible TRALI was relatively high ; 2) patient-related risk factors included higher ASA scores, respiratory comorbidity, lower admission hemoglobin, platelet count, and prothrombin time; 3) surgery-related risk factors included longer duration of surgery and CPB; 4) transfusion related risk factors included higher total number of blood units, RBC, plasma, and platelet units; and 5) measuring PaO_2_/FiO_2_ ratio for an extended period of time post-transfusion helped detect lung injury.

This study showed that worsening respiratory status in cardiac surgery patients categorized as possible TRALI within 24 hours of transfusion occurred in 12.2% of cases, which is higher than previously observed in general hospital population (24-26). The strength of this study lies in its prospective design, whereas most other reports on this subject are retrospective and comprise reports to blood banks on general hospital population (24,26,27).

The incidence of possible TRALI in this study was comparable to studies on TRALI in non surgical critically ill patients. For example, Gajic et al (19) reported on respiratory worsening in 8% of patients who developed ALI within 6 hours of transfusion, while Benson et al reported a 15% incidence of TRALI in critically ill patients with end-stage liver disease (28). On the other hand, it was higher than 2.4% incidence in cardiac surgery patients reported by Vlaar et al (29-31). This difference could be ascribed to different monitoring time, ie, 6 hours in the study by Vlaar et al vs 24 hours in our study. The prolonged monitoring time to up to 24 hours could be perceived as a strong point of the present study. The usage of the expanded definition of TRALI (21) to find “delayed TRALI syndrome” cases is supported by the fact that a considerable proportion of patients (10/32) developed respiratory worsening after standard 6-hour time frame. Therefore, we suggest monitoring for possible TRALI in cardiac surgery patients for more than the standard 6 hours (10).

Similar to studies in critically ill patients (19) and cardiac surgery patients (29-31), in our study more severely ill patients were at a higher risk of possible TRALI. Gajic et al demonstrated worsening of the existing ALI after transfusion in 11.6% of patients (19), which is in line with our observation that pre-transfusion respiratory comorbidity poses a risk for possible TRALI. This fact indicates that physicians should reconsider using transfusion therapy in patients with impaired respiratory function.

Our data on the duration of surgery and CPB as risk factors for possible TRALI are similar to the findings of Vlaar et al, who showed the risk of TRALI to be associated with a longer clamp-, pump-, and surgery time (29,31). A proportion of alterations in lung function may be attributed to surgical factors (eg, hemidiaphragm paresis with concomitant atelectasis, wound pain, etc). There are multiple possible pathophysiological mechanisms by which CPB causes lung injury, eg, endothelial cell swelling, plasma, and protein extravasation into the interstitial tissue, release of proteolytic enzymes, congestion of the alveoli with plasma and RBC, inflammatory reaction, and neutrophil priming (32).

In our study, respiratory worsening in cardiac surgery patients was associated with RBC, plasma, and platelet transfusion in a dose-dependent manner. This is consistent with previous studies in cardiac critically ill patients (29,31). The Blood Banks in Croatia follow the policy of male exclusive plasma as a strategy to reduce the risk of TRALI. However, in critically ill patients even a low volume of antibody titer can be sufficient to initiate TRALI (15).

Clinical diagnosis of TRALI is made by exclusion of other differential diagnoses. Differential diagnoses were established on patient history, physical examination, echocardiogram, electrocardiogram, biomarkers of myocardial injury, chest radiographs, fluid balance, and data from hemodynamic invasive monitoring. Binary logistic regression analysis confirmed that a higher total number of transfused blood units and longer duration of CPB were the risk factors for possible TRALI, which is in accordance with the studies by Vlaar et al in cardiac surgery patients (29,31).

Our study showed the Pao_2_/FiO_2_ ratio was more sensitive than SaO_2_ pulse oximetry measurement for diagnosis of ALI (as a prerequisite for diagnosis of possible TRALI). Therefore, we suggest that clinicians observe PaO_2_/FiO_2_ ratio after transfusion. Their attention should be focused on the patient group where “paradoxical” worsening in the measured PaO_2_/FiO_2_ values develops post-transfusion to identify patients that are more likely to develop possible TRALI.

Our study had some limitations. The results reflect a single center cardiothoracic surgical ICU in a tertiary care hospital, and although internal validity may have been high, external validation was limited and the sample size was relatively small. Moreover, our data could not be compared with other cardiac surgery centers in Croatia. Therefore, a larger multicenter study should be conducted. Another potential drawback is that monitoring for TRALI lasted for up to 24 hours post-transfusion and missed some mild cases that developed later. Due to organizational and economic reasons during the study period, laboratory testing for possible TRALI was limited, which is why it was considered that possible TRALI was a clinical diagnosis for which no exact confirmatory tests were available.

In conclusion, to our knowledge, this is the first study that reported that the post-transfusion PaO_2_/FiO_2_ ratio (considered a marker of lung injury) decreased in possible TRALI patients and increased in transfused controls without ALI. It also showed that the total number of transfused blood units and duration of cardiopulmonary bypass were risk factors for possible TRALI. In order to identify and prevent more possible TRALI cases, clinicians should proactively do serial monitoring of respiratory worsening by measuring PaO_2_/FiO_2_ ratio for an extended period of time (24 hours) post-transfusion in cardiac surgery patients.
